# Disease-Modifying Effects of Vincamine Supplementation in *Drosophila* and Human Cell Models of Parkinson’s Disease Based on *DJ-1* Deficiency

**DOI:** 10.1021/acschemneuro.3c00026

**Published:** 2023-06-08

**Authors:** Francisco José Sanz, Cristina Solana-Manrique, Nuria Paricio

**Affiliations:** † Departamento de Genética, Facultad de Ciencias Biológicas, Universidad de Valencia, Burjassot 46100, Spain; ‡ Instituto Universitario de Biotecnología y Biomedicina (BIOTECMED), Universidad de Valencia, Burjassot 46100, Spain; § Departamento de Fisioterapia, Facultad de Ciencias de La Salud, Universidad Europea de Valencia, Valencia 46010, Spain

**Keywords:** Drosophila, DJ-1, Parkinson’s disease, vincamine, therapeutic compounds, oxidative stress, voltage gated Na^+^ channels, nutraceuticals

## Abstract

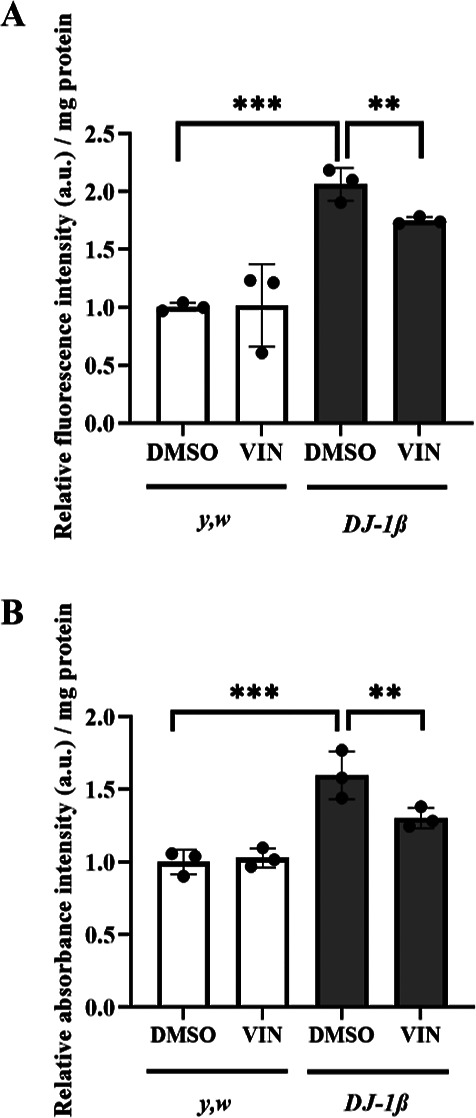

Parkinson’s disease (PD) is an incurable neurodegenerative disorder caused by the selective loss of dopaminergic neurons in the *substantia nigra pars compacta*. Current therapies are only symptomatic and are not able to stop or delay its progression. In order to search for new and more effective therapies, our group carried out a high-throughput screening assay, identifying several candidate compounds that are able to improve locomotor ability in *DJ-1β* mutant flies (a *Drosophila* model of familial PD) and reduce oxidative stress (OS)-induced lethality in *DJ-1*-deficient SH-SY5Y human cells. One of them was vincamine (VIN), a natural alkaloid obtained from the leaves of *Vinca minor*. Our results showed that VIN is able to suppress PD-related phenotypes in both *Drosophila* and human cell PD models. Specifically, VIN reduced OS levels in PD model flies. Besides, VIN diminished OS-induced lethality by decreasing apoptosis, increased mitochondrial viability, and reduced OS levels in *DJ-1*-deficient human cells. In addition, our results show that VIN might be exerting its beneficial role, at least partially, by the inhibition of voltage-gated sodium channels. Therefore, we propose that these channels might be a promising target in the search for new compounds to treat PD and that VIN represents a potential therapeutic treatment for the disease.

## 1 Introduction

Parkinson’s disease (PD) is a progressive and incurable neurological disorder caused by the selective loss of dopaminergic (DA) neurons in the *substantia nigra pars compacta*, which leads to reduced dopamine levels in the striatum.^1^ However, alterations in other neurons as well as in other brain regions have also been found.^2−4^ Neurodegeneration in PD is the result of the combination of processes occurring inside and/or outside the cells. Although its etiopathogenesis remains poorly understood, several works have suggested that mitochondrial alterations, protein misfolding and aggregation, autophagy defects, inflammation, increased oxidative stress (OS) levels, calcium dyshomeostasis, and metabolic alterations might play an important role in the development of the disease.^5−9^ PD is characterized by a range of motor symptoms including bradykinesia, postural instability, and resting tremor, among others. Besides, PD is cursed with non-motor symptoms like mood alterations, sleep disturbances, or even dementia, which significantly reduce patients’ quality of life.^8,10,11^

Current options to treat PD are limited and are mainly based on restoration of dopamine levels in the striatum.^12^ These approaches represent the standard treatment for motor symptoms, but they are not able to halt or delay the progression of the disease.^10,12^ PD is the second most common neurodegenerative disease affecting 1–2% of people over the age of 65, a percentage expected to increase in the near future.^8,10^ In fact, PD is currently the fastest growing neurological disorder in the world.^13^ Therefore, there is an urgent unmet medical need for the identification and development of novel and effective therapies to treat this disease. Several experimental approaches are being used to achieve these goals like gene therapy, immunotherapy, the use of neurotrophic factors, stem cell therapy, and the design of high-throughput screening (HTS) platforms and drug repurposing strategies,^13−18^ among others. In this scenario, we have recently performed an in vivo HTS assay aimed to identify new potential candidate compounds to treat PD, using a *Drosophila* model of the disease based on inactivation of the *DJ-1β* gene (the fly ortholog of human *DJ-1*, a gene involved in familial PD cases).^17^*DJ-1β* mutant flies exhibit several PD-related phenotypes such as reduced lifespan, locomotor defects, as well as increased OS levels.^19−21^ Among the 1120 drugs included in the Prestwick Chemical Library, we identified 10 compounds with an ability to not only suppress motor defects of PD model flies but also reduce OS-induced lethality in *DJ-1*-deficient SH-SY5Y human cells; therefore, these drugs represent promising therapeutic agents for PD.^17^ One of the compounds identified was vincamine (VIN) (referred to as compound B in that study^17^), a natural alkaloid obtained from *Vinca minor*, a species of flowering plant commonly known as lesser or dwarf periwinkle.^22^ Several studies have indicated the potential role of nutraceuticals, such as VIN as well as its semi-synthetic derivative vinpocetine, to target the underlying neurodegenerative processes of PD.^23^ VIN exhibits antioxidant and anti-inflammatory activities, and may work through several mechanisms of action. It is a phosphodiesterase (PDE) I inhibitor, a blocker of voltage-gated sodium (Na^+^) channels (VGNCs), and a GPR40 agonist.^24−26^ VIN is commercially available in the United States as a health-care product with nootropic function and exerts a beneficial effect in different brain-associated disorders in aged patients, like vertigo, memory disturbances, headache, and transient ischemic deficits.^27^ In addition, it enhances cerebral blood flow and glucose uptake, and it is also prescribed to treat memory deficits and cognitive impairments in Alzheimer’s disease (AD) patients.^24^ However, VIN has been barely tested in animal models as a candidate compound to treat PD. Only a recent study has shown that VIN administration reduced motor defects and OS levels in a haloperidol-induced rat PD model and exerted an anti-inflammatory effect.^25^

In this work, we have evaluated the therapeutic potential of VIN in several PD models. Our results have demonstrated that VIN suppressed PD-relevant phenotypes in *Drosophila* and human cell PD models based on *DJ-1* deficiency such as high OS levels, overactivation of the pro-apoptotic JNK pathway, and mitochondrial dysfunction. In addition, we have found that VIN could be exerting a neuroprotective effect through the blockage of VGNCs, thus indicating that these channels might be a promising target for the identification of new treatments for PD.

## 2 Results and Discussion

### 2.1 VIN Reduces OS Levels in *DJ-1β* Mutant Flies

**Figure 1:**
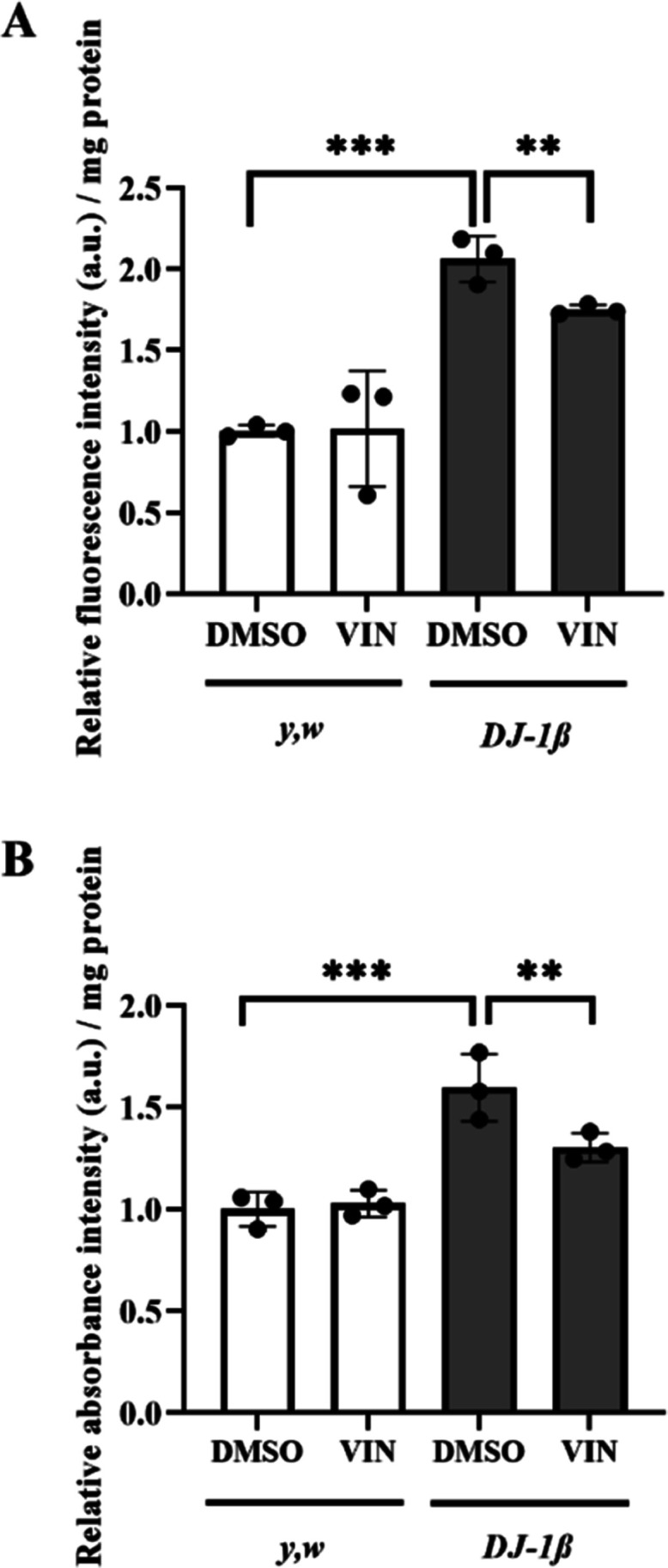
Effect of VIN on oxidative stress marker levels in *DJ-1β* mutant and *y,w* control flies. (A) H_2_O_2_ levels in control and *DJ-1β* mutant flies treated with 10 μM VIN were quantified using the Amplex Red H_2_O_2_ Kit (Invitrogen). (B) Protein carbonylation levels in *DJ-1β* mutant and control flies treated with 10 μM VIN were analyzed by absorbance. In all cases, data were expressed as arbitrary units (a.u.) per mg of protein, and the results were normalized to data obtained from flies cultured in vehicle medium (0.1% DMSO). Error bars show s.d. from at least three replicates and three independent experiments (**P < 0.01; ***P < 0.001).

Among the numerous functions ascribed to the DJ-1 protein, it stands out for its essential role in the defense against OS.^28^ Increased OS levels are observed in brains of PD patients,^6^ which suggests that they play an important role in the development of the disease.^6,29^ According to this, previous studies performed by our group have already shown that compounds with antioxidant properties were able to suppress PD-related phenotypes in fly and cell models of the disease based on *DJ-1* deficiency.^17,29,30^ As mentioned above, VIN was one of the lead compounds identified in an in vivo HTS assay using *DJ-1β* mutant flies (a *Drosophila* PD model) and validated in *DJ-1*-deficient neuron-like cells.^17^ Previous studies have shown that VIN administration in control rats resulted in a significant reduction of brain iron levels.^22^ Iron appears to accumulate in high concentration in neurodegenerative diseases (NDs), such as PD or AD, thus contributing to OS and in turn contributing to neurodegeneration.^31^ Since age-linked NDs are characterized by a disturbance in trace element levels in the brain, it was suggested that VIN might exert a beneficial effect in aged people by decreasing OS.^22^ It was also shown that VIN was able to reduce Aβ-induced cytotoxicity in PC12 cells by decreasing the concentrations/activities of a variety of OS indicators.^32^ As reported previously, *DJ-1β* mutants exhibited high reactive oxygen species (ROS) levels and increased protein carbonylation (a post-translational modification caused by high ROS levels) when compared to control flies.^19,30^ Indeed, we demonstrated that they had a causative role in motor deficits exhibited by PD model flies.^29^ In such a scenario, we decided to evaluate the levels of OS indicators in the PD model and control flies after VIN supplementation. As shown in Figure 1, we found that *DJ-1β* mutant flies treated with 10 μM VIN during development and 5 days after eclosion presented a significant reduction of H_2_O_2_ (an element of the total ROS pool) and of protein carbonylation levels compared to flies treated with vehicle (0.1% DMSO) (Figure 1). No significant differences were observed between treated and untreated control flies. Therefore, our results indicate that VIN treatment exerts an antioxidant effect in *DJ-1β* mutant flies. Interestingly, a recent study has demonstrated that VIN administration also reduced motor defects and OS levels in a haloperidol-induced rat PD model.^25^ Taken together, these results indicate the therapeutic potential of VIN in animal models of PD.

### 2.2 VIN Increases Viability of *DJ-1*-Deficient Human Cells by Reducing JNK Signaling Activation

**Figure 2:**
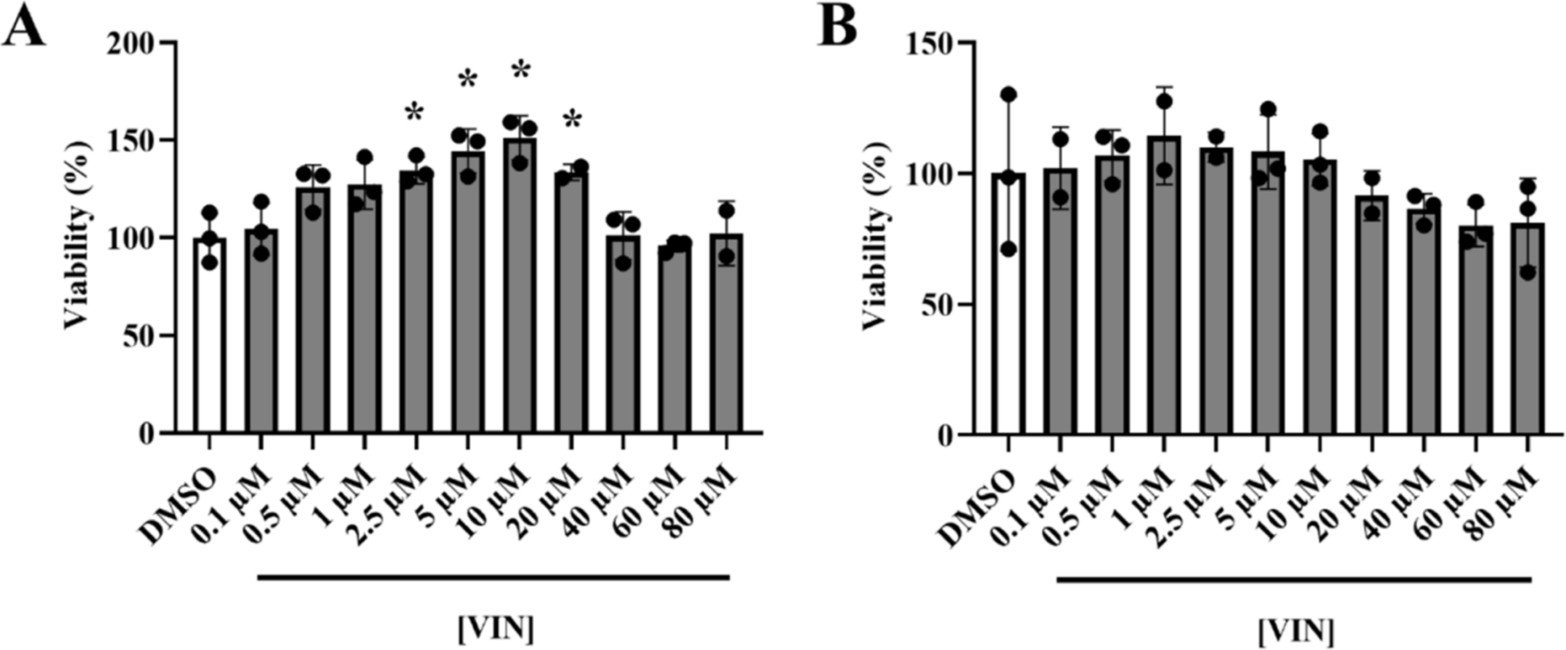
Effect of VIN on the viability of *DJ-1*-deficient and control SH-SY5Y cells. MTT assays were performed to measure the viability of (A) *DJ-1*-deficient and (B) *pLKO.1* control cells grown under OS conditions (induced with 100 μM H_2_O_2_) either treated with vehicle (0.1% DMSO) or with different concentrations of VIN (0.1–80 μM). The results were normalized to data obtained from vehicle-treated cells. Error bars show s.d. from three independent experiments in which three biological replicates were used (*P < 0.05).

Although *Drosophila* is an outstanding model organism in the search of new treatments for human diseases, candidate compounds identified in flies have to be validated in mammalian models.^17,33^ It has been previously reported that viability of *DJ-1*-deficient human neuroblastoma cells was reduced when cultured under OS conditions.^29^ We already demonstrated that pretreatment with 10 μM VIN significantly attenuated OS-induced death in *DJ-1*-deficient cells.^17^ Therefore, we decided to further analyze the effect of VIN in cell viability by pretreating such cells with different concentrations of the compound from 0.1 to 80 μM. Our results showed that VIN exerted neuroprotective effects in a range of 2.5–20 μM, with10 μM being the most effective concentration (Figure 2A). Thus, we used this concentration in subsequent experiments performed in human cells. Furthermore, as shown in Figure 2B, VIN did not have a detrimental effect in *pLKO.1* control cells at the same concentrations, indicating that its effect may depend on *DJ-1* deficiency (Figure 2B).

**Figure 3:**
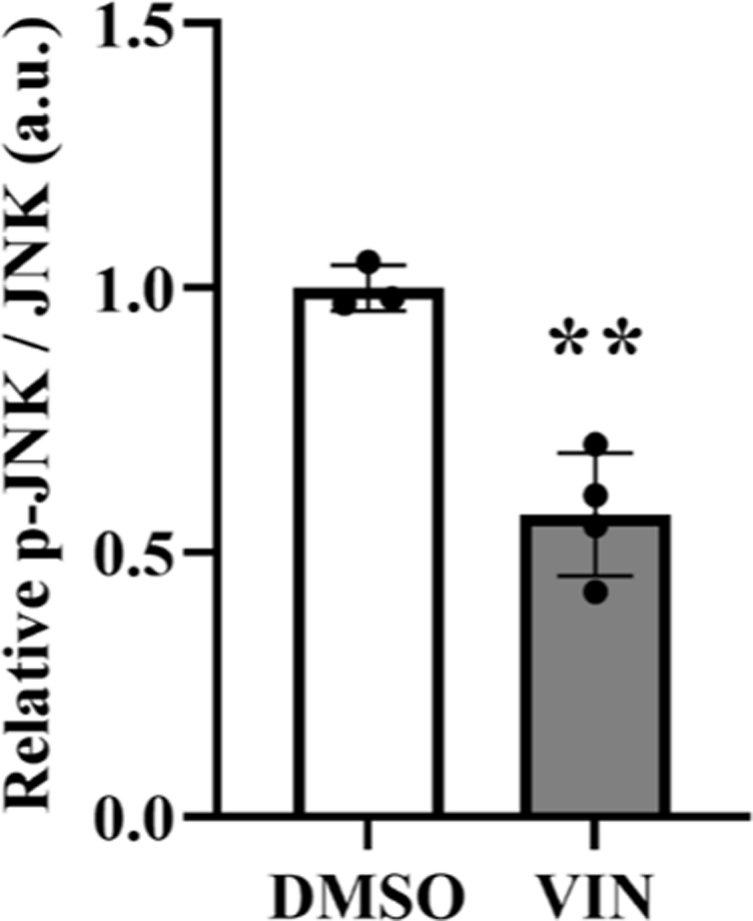
Effect of VIN on JNK pathway activity in *DJ-1*-deficient SH-SY5Y cells. Western blot analyses were carried out using antibodies against JNK, and *p*-JNK in *DJ-1*-deficient cells cultured under OS conditions (induced with 100 μM H_2_O_2_) and treated with 10 μM VIN or vehicle (0.1% DMSO). The relative ratio of *p*-JNK/JNK was analyzed by densitometry. The results were normalized to data obtained from vehicle-treated *DJ-1*-deficient cells and expressed as arbitrary units (a.u.). Error bars show s.d. from four biological replicates (**P < 0.01).

PD is caused by the loss of DA neurons; however, the reason of this neurodegeneration is still unknown.^34^ Among the processes that might lead to neuronal death, we find apoptosis,^35,36^ a highly regulated process where the JNK protein plays a key role.^36^ It has been reported that *DJ-1*-deficient SH-SY5Y cells present high levels of JNK phosphorylation, which activates the JNK signaling pathway and promotes cell death.^17,36,37^ In order to evaluate if VIN could be exerting its neuroprotective effect through apoptosis reduction, we performed a Western blot assay to study its effect on JNK phosphorylation. We found that JNK phosphorylation levels were significantly reduced in *DJ-1*-deficient cells pretreated with 10 μM VIN compared to cells pretreated with vehicle (0.1% DMSO) (Figures 3, S1), thereby reducing the activity of the pro-apoptotic JNK pathway as well as increasing viability of *DJ-1*-deficient cells. In agreement with our results, it was demonstrated that VIN exerted a protective effect in rat livers treated with tamoxifen, a compound that induces cell death.^38^ Indeed, rats treated with VIN showed a decrease of tamoxifen-induced hepatic cell injury via suppressing OS and reducing JNK phosphorylation.^38^

### 2.3 VIN Enhances Mitochondrial Viability in *DJ-1*-Deficient Human Cells

**Figure 4:**
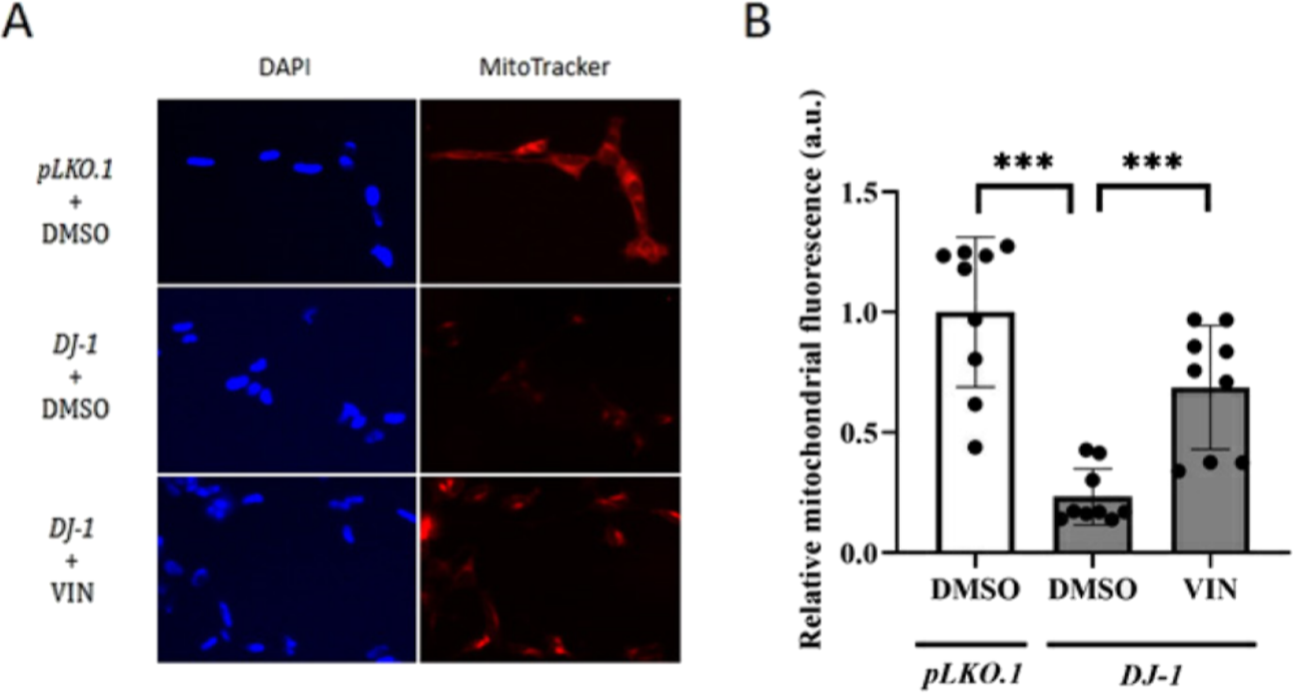
Effect of VIN on mitochondrial viability in SH-SY5Y cells. (A) Representative fluorescence microscopy images of *pLKO.1* control cells pretreated with vehicle (0.1% DMSO), and *DJ-1*-deficient cells either pretreated with vehicle (0.1% DMSO) or with 10 μM VIN, stained with the mitochondrial dye MitoTracker Red FM and the nuclear dye DAPI (blue). Cells stained were *pLKO.1* control cells pretreated with vehicle (0.1% DMSO), and *DJ-1*-deficient cells either pretreated with vehicle (0.1% DMSO) or with 10 μM VIN. (B) Graphical representation of Mitotracker Red FM fluorescence quantification from (A). At least 10 images of each strain and treatment were analyzed. Results were normalized to data obtained from vehicle-treated *pLKO.1* control cells and expressed as arbitrary units (a.u.). Error bars show s.d. from nine independent experiments (***P < 0.001).

Mitochondrial dysfunction plays an important role in PD.^39^ In fact, many of the genes involved in familial PD cases are functionally associated with mitochondria; this highlights its relevance in PD development.^40^ Specifically, loss of *DJ-1* function has been linked to a reduction of mitochondrial mass as well as to alterations in the morphology and function of this organelle.^37,41,42^ Interestingly, the activation of JNK signaling was also related to the onset of mitochondrial dysfunction.^43^ Previous studies carried out by our group demonstrated that *DJ-1*-deficient human cells presented a reduction of mitochondrial viability compared to *pLKO.1* control cells.^17^ Therefore, we decided to evaluate whether VIN supplementation could increase such viability in mutant cells using the MitoTracker Red FM dye. As expected, we found that *DJ-1*-deficient cells presented a significant reduction in the active mitochondrial mass compared to *pLKO.1* control cells (Figure 4A,B). Our results also showed that VIN pretreatment of *DJ-1*-deficient cells enhanced mitochondrial viability compared to those treated with vehicle (0.1% DMSO) (Figure 4A,B).

**Figure 5:**
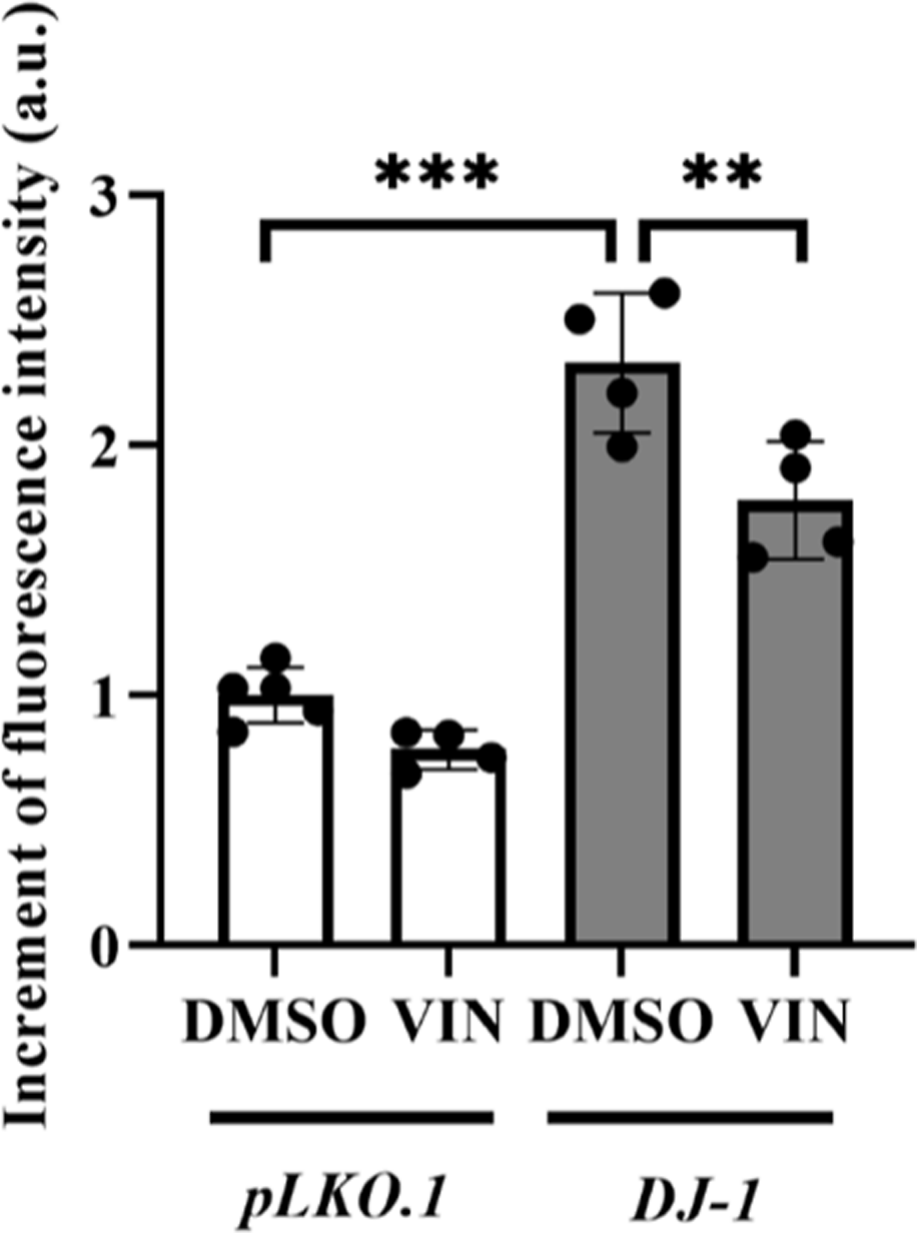
Effect of VIN on intracellular ROS levels in *DJ-1*-deficient SH-SY5Y cells. Intracellular ROS levels in *pLKO.1* and *DJ-1*-deficient cells treated either with vehicle (0.1% DMSO) or with 10 μM VIN were analyzed using the fluorescence dye dihydroethidium (Invitrogen). Results were normalized to data obtained from vehicle-treated *pLKO.1* control cells and expressed as arbitrary units (a.u.). Error bars show s.d. from four biological replicates (**P < 0.01; ***P < 0.001).

Mitochondria are one of the main sources of ROS, which are by-products of their normal metabolism and homeostasis. Thus, alterations in mitochondrial function may lead to an increase of ROS levels above a toxicity threshold resulting in potentially unwanted oxidative consequences and even cell death.^44^ For instance, it has been found that mitochondrial damage in PD might be caused by complex I of the electron transport chain (ETC) dysfunction,^40,45^ a complex to which the DJ-1 protein directly binds.^46^ In addition, several toxins (rotenone, paraquat or MPTP) able to inhibit its activity are commonly used to generate animal and cell models of idiopathic PD.^40,47^ Since PD model cells showed a decrease in active mitochondria, we aimed to study the consequence of that reduction in ROS levels. For doing so, we used the dihydroethidium fluorescence dye to quantify intracellular ROS levels in *DJ-1*-deficient and control cells.^48^ We found that *DJ-1*-deficient cells presented higher ROS levels than *pLKO.1* control cells (Figure 5). In addition, we confirmed that *DJ-1*-deficient cells supplemented with VIN showed a reduction in intracellular ROS levels compared to vehicle-treated cells (0.1% DMSO) (Figure 5).

Taken together, our results support the therapeutic potential of VIN in *DJ-1*-deficient cells to ameliorate PD-associated mitochondrial dysfunction and the consequent increase in ROS levels, as shown in other disease models.^25,38^

### 2.4 Veratridine, a VGNC Activator, Reduces the Neuroprotective Effect of VIN in *DJ-1*-Deficient Human Cells

As mentioned previously, VIN is a compound with multiple mechanism of actions. Several studies have shown that it is a PDE1 inhibitor, a GPR40 agonist, and a blocker of VGNCs.^24−26^ The effect of PDE inhibitors has been widely studied in several PD models. For example, it was demonstrated that PDE1 inhibitors induced the expression of genes related to neuronal plasticity, neurotrophic factors, as well as molecules with neuroprotective function.^49^ Since PDE inhibitors have been already proposed as promising PD therapeutic compounds,^50^ we decided to evaluate whether VIN could also be exerting its neuroprotective effect in PD models based on *DJ-1* deficiency through a different mechanism of action, such as VGNC inhibition. These channels play a vital role in excitable cells (like cardiomyocytes and neurons) to generate and propagate action potentials. Their functional deficits lead to epilepsy, a brain disorder characterized by seizures and convulsions.^51^ Interestingly, a recent study has shown that VGNCs may play a substantial role in the onset of cognitive defects in PD model rats.^52^

**Figure 6:**
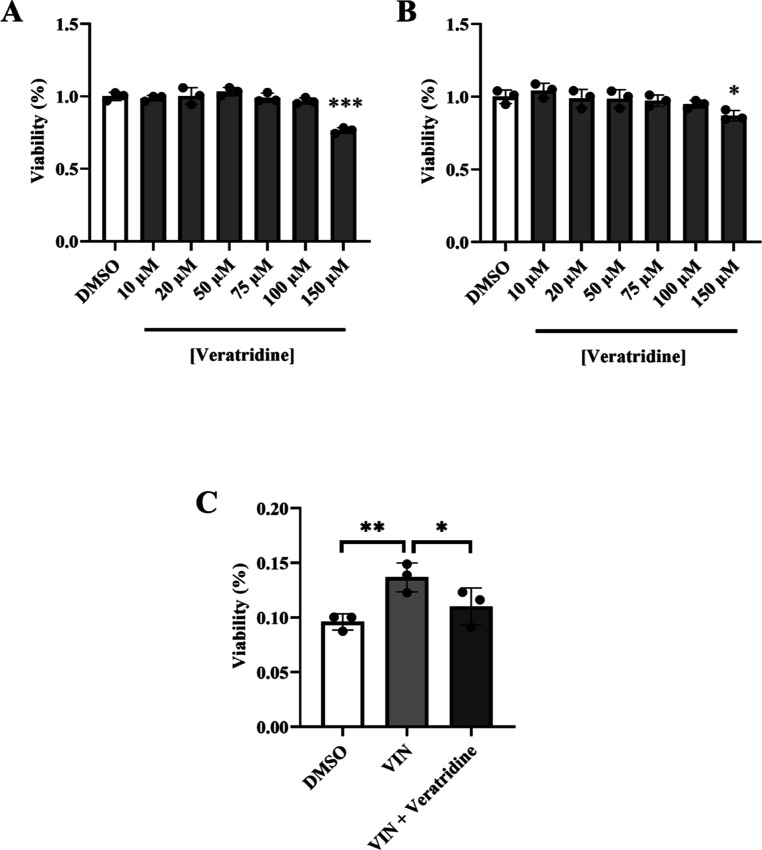
Effect of veratridine on the viability of *DJ-1*-deficient SH-SY5Y cells treated with VIN under OS conditions. MTT assays were performed to measure the viability of (A) *pLKO.1* control and (B) *DJ-1*-deficient cells subjected to OS (induced with 100 μM H_2_O_2_) and either treated with vehicle (0.1% DMSO) or with different veratridine concentrations (1–150 μM). In both cases, results were normalized to data obtained in vehicle-treated cells. (C) Viability of *DJ-1*-deficient cells under OS conditions and treated with vehicle (0.1% DMSO), 10 μM VIN or 10 μM VIN plus 100 μM veratridine. Results were normalized to data obtained from vehicle-treated *DJ-1*-deficient cells. In all cases, error bars show s.d. from three independent biological replicates (*P < 0.05; **P < 0.01; ***P < 0.001).

In such a scenario, we aimed to determine if VIN could be exerting a neuroprotective effect in *DJ-1*-deficient cells through inhibition of VGNCs. For doing so, we tested whether veratridine, an alkaloid able to induce persistent activation of these channels,^53^ could affect VIN-mediated neuroprotection. First, we tested different concentrations of veratridine in a range of 10–150 μM in order to identify the maximum concentration of the compound that did not exert a detrimental effect in cell survival under OS conditions. Our results showed that viability of *pLKO.1* control and *DJ-1* mutant cells was significantly reduced under OS conditions when using 150 μM of veratridine (Figure 6A,B); in contrast, viability was not affected with lower concentrations of the compound. Therefore, we decided to use 100 μM of veratridine to test its effect on cells treated with VIN. Our results showed that viability of VIN-treated *DJ-1*-deficient cells was significantly reduced after veratridine pretreatment (Figure 6C). Thus, these results suggest that VIN might be exerting a neuroprotective effect, at least partially, through the inhibition of VGNCs. Supporting our results, it was recently reported that VGNCs were overexpressed in a 6-OHDA-induced rat model of PD, and that phenytoin, a VGNC blocker, improved motor and cognitive abilities in that model.^54^ In addition, it was found that treatment with RS100642, a VGNC blocker, reduced levels of OS markers in a rat model of breast cancer;^55^ therefore, VGNC inhibition could play a role in the defense against OS. Overall, these results suggest that VGNCs represent a potential target in the search of novel and more efficient treatments for PD.

## 3 Materials and Methods

### 3.1 *Drosophila* Stocks and Drug Treatment

Fly stocks used in this study were *y^1^, w^1118^* (hereafter called *y,w*) from the Bloomington *Drosophila* Stock Center, and the *DJ-1β^ex54^* strain^56^ (hereafter called DJ-1β). Flies were maintained and cultured at 25 °C in standard *Drosophila* medium containing sucrose, yeast, cornmeal, soybean flour, agar, propionic acid, ethanol, and propil-*p*-hydroxybenzoate. In treatments, flies were cultured on standard medium containing 0.1% dimethyl sulfoxide (DMSO) (untreated flies) or supplemented with 10 μM VIN (Tebubio, T1286).

### 3.2 Quantification of H_2_O_2_ Levels and Protein Carbonyl Group Formation in Whole Fly Extracts

H_2_O_2_ and protein carbonylation levels were measured in 5-day-old *DJ-1β* mutant female flies treated with vehicle (0.1% DMSO) or with 10 μM VIN. Quantification of H_2_O_2_ levels was carried out in fly extracts using the Amplex Red Hydrogen Peroxide/Peroxidase Assay Kit (Invitrogen) as described previously in ref (29). Protein carbonyl groups were quantified in female fly extracts using 2,4-dinitrophenyl hydrazine derivatization as described previously in ref (17). All experiments were carried out using three biological replicates and three technical replicates per sample.

### 3.3 SH-SY5Y Cells Culture and Drug Treatment

In this study, we used *pLKO.1* control and *DJ-1*-deficient SH-SY5Y neuron-like cells previously generated by our group.^29^ Cells were cultured at 37 °C and 5% CO_2_ in selective growth medium consisting of Dulbecco’s Modified Eagle Medium/Nutrient Mixture F-12 (DMEM/F12) (Biowest) and supplemented with 10% (v/v) fetal bovine serum (Capricorn), 1% non-essential amino acids, 100 mg/mL penicil/streptomycin (Labclinics), and 2 μg/mL puromycin (Labclinics). Viability of cells treated with VIN, the VNGC activator veratridine (Santa Cruz Biotechnology, sc-201075), or 0.1% DMSO (vehicle) was evaluated using a MTT assay (Sigma-Aldrich) as described in.^29^ To evaluate if veratridine could impair the beneficial effect of our candidate compound, cells were pretreated for 2 h with 150 μM of the VNGC activator before the addition of VIN. Subsequently, viability assays were performed as described in ref (29). All experiments were carried out using three biological replicates and three technical replicates per sample.

### 3.4 Mitochondrial Viability

Mitochondrial viability of *DJ-1*-deficient and *pLKO.1* control cells was evaluated using the MitoTracker Red FM (Invitrogen) fluorescence dye as described in ref. 17. The cells were grown under OS conditions (induced with 100 μM H_2_O_2_) and treated either with 10 μM VIN or with vehicle (0.1% DMSO). Images were obtained using a fluorescence microscope (Leica DMI3000 B), and ImageJ software (NIH) was employed to analyze them. All experiments were carried out using nine biological replicates per sample.

### 3.5 Western Blot Analyses

Protein extraction and Western blot of lysates from *DJ-1*-deficient SH-SY5Y cells grown under OS conditions and treated with 10 μM VIN or with vehicle (0.1% DMSO) were performed as described previously in ref (57). Antibodies used in this study were anti-JNK (1:1000, Cell Signaling, #9252), anti-phospho-JNK (Thr183/Tyr185) (1:1000, Cell Signaling, #4668P), and anti-rabbit HRP-conjugated (1:5000, Sigma, 12–348). Quantifications of protein levels were performed with an ImageQuant LAS 4000mini Biomolecular Imager (GE Healthcare), and images were analyzed with ImageJ software (NIH). All experiments were carried out using four biological replicates.

### 3.6 Quantification of ROS Levels in Human SH-SY5Y Cells

Quantification of ROS levels in *DJ-1*-deficient and *pLKO.1* control cells treated with 10 μM VIN or with vehicle (0.1% DMSO) was carried out using the dihydroethidium fluorescence dye (Invitrogen), and following a protocol adapted from ref (58). Briefly, 1.8 × 10^4^ cells/well were seeded in a black 96-well plate and incubated for 24 h at 37 °C and 5% CO_2_. Subsequently, they were incubated with 100 μM H_2_O_2_ for 3 h under the same conditions. Finally, dihydroethidium was added to each well at a final concentration of 10 μM. Fluorescence was measured at 0 and 30 min at a wavelength of excitation and emission of 540 nm and 595 nm, respectively, in an Infinite 200 PRO reader (Tecan). ROS levels of each sample were calculated using the following formula: [(*F*_30min_–*F*_0min_)/*F*_0min_] × 100. All experiments were carried out using four biological replicates.

### 3.7 Statistical Analyses

The significance of differences between means was assessed using a *t*-test when two experimental groups were analyzed. In experiments in which more than two experimental groups were used, the statistical analysis was made using the ANOVA test and Tukey’s post hoc test. Differences were considered significant when *P* < 0.05. Data are expressed as means ± standard deviation (s.d.).

## 4 Conclusions

In this work, we have evaluated the therapeutic potential of VIN, a natural alkaloid, as PD treatment using preclinical models of the disease. This study has helped to shed light on the molecular mechanism of the drug’s action and to identify how VIN can mostly exert its beneficial effect in PD models. Indeed, we have demonstrated that VIN is able to ameliorate PD-related phenotypes in *Drosophila* and human cell models based on DJ-1 inactivation. Specifically, VIN was able to increase viability in *DJ-1*-deficient SH-SY5Y human cells by reducing apoptosis, to increase mitochondrial viability, and to reduce the level of OS indicators. Taken together, these results clearly show the disease-modifying effect of VIN and allow us to consider this compound as a promising PD therapy. In addition, we have demonstrated that VIN might exert, in part, its neuroprotective effect through VGNC inhibition, thus proposing these channels as potential targets in the discovery of new and more effective therapeutics to treat PD.

Although studies with VIN in PD models are scarce, the effect of vinpocetine (a VIN derivative with the same mechanisms of action) has been thoroughly tested in animal models of PD and other human diseases.^59^ For example, this compound was shown to increase cerebral blood flow, hence improving O_2_ and glucose uptake by neurons, which leads to an increase of ATP levels.^60^ Besides, it has anti-inflammatory effects in PD patients,^61^ and it was shown to reduce motor defects, cognitive alterations, OS levels, and DA neurodegeneration as well as to increase dopamine levels in mouse and rat PD models.^62,63^ Our results clearly support further investigation of VIN as a promising PD treatment. VIN is non-toxic, can cross the blood–brain-barrier, and is currently used as a dietary supplement; therefore, it is an outstanding candidate for clinical trials in PD patients. Moreover, it has multiple beneficial properties not only for neural disorders but also as an anticancer agent.^64^ All these properties make VIN a promising drug for future exploration to identify the precise mechanisms underlying its beneficial effect in PD.

## Supporting Information Available

JNK and P-JNK expression in *DJ-1*-deficient SH-SY5Y cells treated with VIN; representative western blot using anti-JNK and anti-P-JNK antibodies in protein extracts from DJ-1-deficient cells either treated with 10 μM VIN or with vehicle (0.1% DMSO) (PDF)

## Abbrevations

AD: Alzheimer’s disease
DA: dopaminergic
ETC: electron transport chain
HTS: high-throughput screening
ND: neurodegenerative disease
OS: oxidative stress
PD: Parkinson’s disease
PDE: phosphodiesterase
ROS: reactive oxygen species
VGNC: voltage gated Na ^+^ channels
VIN: vincamine.
