# Supplementary figures and images for: Disease-Modifying Effects of Vincamine Supplementation in Drosophila and Human Cell Models of Parkinson’s Disease Based on DJ-1 Deficiency
Source: ACS Chem Neurosci. 2023 Jun 8;14(12):2294–301. doi: 10.1021/acschemneuro.3c00026 (PMC10288506; doi:10.1021/acschemneuro.3c00026)

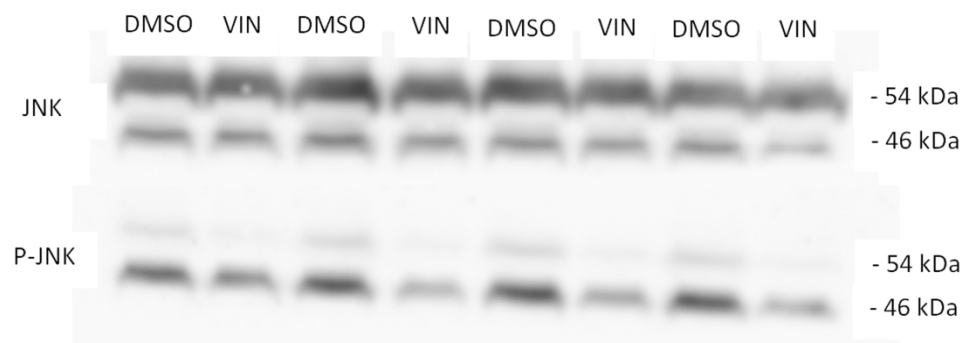

170x62mm (600 x 600 DPI)

Supplement: Supplementary file 1 — cn3c00026_si_001.pdf [file cn3c00026_si_001.pdf]
